# Phenological indices of avian reproduction: cryptic shifts and prediction across large spatial and temporal scales

**DOI:** 10.1002/ece3.558

**Published:** 2013-05-21

**Authors:** Philippa Gullett, Ben J Hatchwell, Robert A Robinson, Karl L Evans

**Affiliations:** 1 Department of Animal & Plant Sciences, University of SheffieldSheffield, S10 2TN, UK; 2 British Trust for Ornithology, The NunneryThetford, Norfolk, IP24 2PU, UK

**Keywords:** Fecundity, first egg date, global warming, lay date, microevolutionary change, predation, reproductive window, selection pressure, timing of breeding, trophic mismatch

## Abstract

Climate change-induced shifts in phenology have important demographic consequences, and are frequently used to assess species' sensitivity to climate change. Therefore, developing accurate phenological predictions is an important step in modeling species' responses to climate change. The ability of such phenological models to predict effects at larger spatial and temporal scales has rarely been assessed. It is also not clear whether the most frequently used phenological index, namely the average date of a phenological event across a population, adequately captures phenological shifts in the distribution of events across the season. We use the long-tailed tit *Aegithalos caudatus* (Fig. 1) as a case study to explore these issues. We use an intensive 17-year local study to model mean breeding date and test the capacity of this local model to predict phenology at larger spatial and temporal scales. We assess whether local models of breeding initiation, termination, and renesting reveal phenological shifts and responses to climate not detected by a standard phenological index, that is, population average lay date. These models take predation timing/intensity into account. The locally-derived model performs well at predicting phenology at the national scale over several decades, at both high and low temperatures. In the local model, a trend toward warmer Aprils is associated with a significant advance in termination dates, probably in response to phenological shifts in food supply. This results in a 33% reduction in breeding season length over 17 years – a substantial loss of reproductive opportunity that is not detected by the index of population average lay date. We show that standard phenological indices can fail to detect patterns indicative of negative climatic effects, potentially biasing assessments of species' vulnerability to climate change. More positively, we demonstrate the potential of detailed local studies for developing broader-scale predictive models of future phenological shifts.

## Introduction

Phenology plays a key role in regulating species interactions that can determine population dynamics (Miller-Rushing et al. 2010). Recent climate change has brought about phenological shifts in a wide range of species (Walther et al. 2002; Thackeray et al. 2010), with a particularly well-studied example being the earlier onset of breeding in temperate bird populations in years with warmer spring conditions (Thomas et al. 2001; Charmantier et al. 2008; Both et al. 2009). Species exhibiting larger phenological shifts are typically more resilient to the negative impacts of climate change than those exhibiting more limited phenological advance (Møller et al. 2008; Jones and Cresswell 2010), and predicting future phenological trends would therefore facilitate assessment of species' sensitivity to climate change (Diez et al. 2012). Predictive capacity could be limited by non-linearity in climatic responses, local adaptation, and variation in the capacity to exhibit plastic phenological responses (Primack et al. 2009; Perfito et al. 2012; Porlier et al. 2012), but empirical assessments of the ability of phenological models to predict responses at different spatial or temporal scales are very rare (but see Hodgson et al. 2011) and urgently needed (Diez et al. 2012).

**Figure 1:**
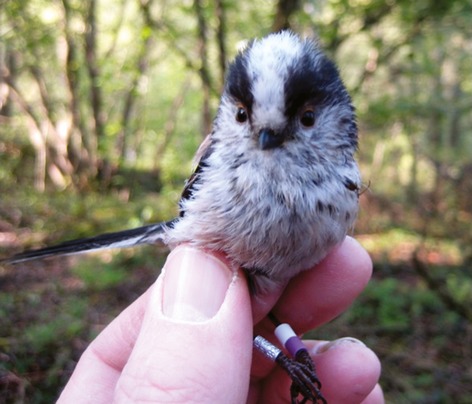
A long-tailed tit *Aegithalos caudatus* in the Rivelin Valley, Sheffield.

**Figure 2:**
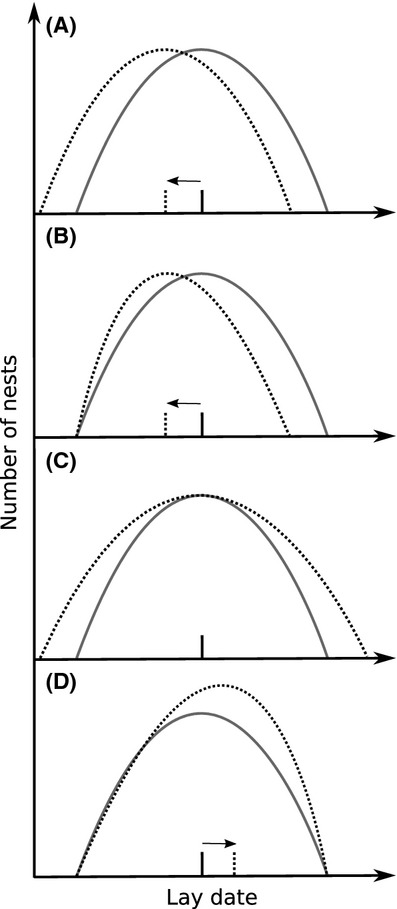
Hypothetical distributions of population lay dates prior to (solid line) and following (dotted line) climate change, showing resultant changes in mean lay date. Numerous responses are possible, but this subset illustrates the problem with using population mean breeding date as a phenological indicator of species' responses to climate change. Mean breeding date can advance when populations: (A) start and end breeding earlier or (B) start at the same time, but end breeding earlier. Mean breeding date may also (C) exhibit no advance when breeding commences earlier if breeding continues for longer, and (D) exhibit changes that are unrelated to climate change, such as a later mean breeding date due to increased predation rates that increase the proportion of renests.

Some of the assumptions underlying the use of phenological indices in the assessment of species' vulnerability to climate change also warrant more detailed empirical testing. Many phenological indices in frequent use are calculated as the mean timing of an event across the focal population. These ‘population mean indices’ are certainly preferable to indices of the timing of first events (Miller-Rushing et al. 2008), which only capture responses of a very limited proportion of the focal population. However, indices of a population's mean timing assume that climate change does not alter the distribution of events within a season. This is not always true; for instance, the mean timing of avian breeding is sensitive to climatic influences on the frequency of second broods (Visser et al. 2003; Husby et al. 2009). There has, however, been insufficient exploration of how climate change alters the distribution of breeding attempts in single-brooded species, and the consequences of this for using phenological indices as indicators of species' sensitivity to climate change. If climate change has equivalent impacts on the timing of breeding initiation and termination then phenological indices of the mean timing of reproduction are robust (Fig. 2A). Different months of the breeding season can, however, exhibit divergent climatic trends that may result in different impacts on initiation and termination (Houghton et al. 2001; Halupka et al. 2008). Consequently, an advance in the population mean lay date could be observed even if the onset of reproduction has not advanced, due to earlier termination of breeding attempts (Dawson 2005; Fig. 2B). In contrast, mean breeding date will not advance, even if onset of breeding has advanced, if the end of the breeding season is delayed by a similar amount (Fig 2C); such lengthening of the breeding season could arise if longer growing seasons (Menzel and Fabian 1999) increase food availability both early and late in the season. Finally, a shift in predation regime could alter the proportion of pairs building repeat nests, thus driving a change in a population's mean lay date that is unrelated to climate change; for example, an increase in nest predation rates is likely to generate more replacement nests later in the season, thus delaying mean breeding dates (Fig. 1D).

Here, we extend previous work that has determined how climate change alters the distribution of breeding attempts in multiple-brooded species by focusing on a single-brooded species. We assess whether population average lay date is a reliable indicator of the distribution of breeding events, and hence of phenological shifts and sensitivity to climate change. We use high-resolution data from an intensive 17-year study of the long-tailed tit *Aegithalos caudatus* in central England, and extensive national scale data collected over a 43-year period. This single-brooded species provides an ideal case study as it exhibits one of the most rapid advances in mean annual lay date among British birds (Baillie et al. 2012). Moreover, in contrast to almost all other avian species subject to intensive phenological studies, the long-tailed tit does not use nest boxes, and thus experiences high rates of nest predation (˜70%), resulting in renests accounting for approximately 40% of nesting attempts per year (Hatchwell et al. 1999). This allows us to determine the nature of associations between the timing and intensity of predation regimes, and phenological indices of the timing of reproduction. We also provide the first empirical assessment of whether locally derived models of avian phenology can be scaled up to predict phenological trends at larger spatial scales and in different time periods. This is an essential first step toward predicting phenological trends under future climate change scenarios.

## Materials and Methods

### Study system

We studied a population of 25–72 pairs (mean 46 pairs) of long-tailed tits in the Rivelin Valley, Sheffield, U.K. (53°23′N, 1°34′W). Long-tailed tits are single brooded, but nest predation rates are high and pairs that fail frequently initiate a second or third renest attempt if there is sufficient time to raise a brood. Renests thus account for around 32% of nests per year (range = 0.26–0.40), and the proportion of renesting showed no temporal trend over the course of the study (*r*^2^_1,13_ = 0.05, *P* = 0.43). The long-tailed tit is a facultative cooperative breeder and some failed breeders help other pairs rather than renesting themselves, particularly if they fail later in the season (MacColl and Hatchwell 2002; Hatchwell et al. 2004). At least 95% of adult birds in the study site are uniquely marked with color-rings each breeding season and all pairs within the study site are monitored and their nests located by observation. A very small proportion (estimated to be <5%) of nesting attempts are not found each year, but through monitoring parental activity it is known that the vast majority of these are short-lived attempts that rapidly fail (Sharp et al. 2008). Nests are monitored approximately every 2 days. In the case of nest failure, the study site is searched intensively for renesting attempts. The date on which the first egg of each clutch is laid (hereafter referred to as first egg date) is recorded so that day 1 corresponds to 1 March, and is accurate to within 1 day for all accessible nests. Inaccessible nests comprise approximately 10% of the data, and their first egg date is estimated by observing the time at which parents stop lining nests, which typically occurs when laying starts, and/or by recording the date when females commence incubation (the last day of egg laying) and assuming a clutch of 10 eggs (the modal clutch size in the study population; Hatchwell et al. 2004). Observations of nestling provisioning and fledging dates suggest that these lay dates for inaccessible nests are generally accurate to ± 2 days.

### Datasets

Data were collected from the local Rivelin population of long-tailed tits during 1995–2011, including first egg dates for 824 nests (559 first nesting attempts and 265 renests) and failure dates for 590 nests (410 first attempts and 180 renests). Data from 2001 were omitted from all analyses because access to the field site was limited by an outbreak of foot and mouth disease, and data from 2003 were excluded in analyses regarding the timing of renesting and termination of breeding due to limited search effort for renests at the very end of the field season in that year. Weather data were obtained for the period 1968–2011 from the Weston Park Meteorological Station, located 5 km from the center of the Rivelin study site. Monthly mean temperature and monthly total precipitation were calculated. These data were very strongly correlated with UK Climate Projections (UKCP) interpolated data (available until 2006, Jenkins et al. 2008) for the 5 km × 5 km grid cell containing the study site (Pearson's correlations with *n* = 11; May precipitation: *rp* = 0.84; all other comparisons: *rp* > 0.96). Food abundance data were also collected at four locations, from a total of 16 trees, within the Rivelin study site by collecting caterpillar frass samples throughout the spring for the period 2009–2012, as caterpillars are the primary food source of long-tailed tits provisioning nestlings. We hence calculated the annual date of peak caterpillar abundance, and assessed the association between peak date and spring weather variables to test the hypothesis that food abundance declines earlier in years with warmer spring temperatures (see Appendix S1 for full methods).

National data were obtained for the period 1968–2010 from the British Trust for Ornithology's (BTO) Nest Record Scheme (NRS; Crick et al. 2003). These nest records include an unknown proportion of first nesting attempts and renests, from various locations throughout the U.K. (mainly England) excluding the Rivelin Valley area. The mean annual lay date was calculated across all records within each year (mean annual sample size = 50; range = 18–123). For weather data, we used Central England Temperature from the HadCET database (Parker et al. 1992), as used in previous analyses of climatic influences on lay dates using nest record card data (Crick et al. 1997; Crick and Sparks 1999).

### Constructing phenological indices

#### Initiation date, renesting date, average lay date

The distribution of first egg dates within each year deviated from a normal distribution so we used median first egg dates as a phenological indicator, although there was a strong correlation between mean and median first egg dates (all attempts: *rp*_14_ = 0.944, *P* < 0.001; first attempts: *rp*_14_ = 0.996, *P* < 0.001; renests: *rp*_13_ = 0.921, *P* < 0.001). Within each year, initiation date was the median of first egg dates from first attempts; renesting date was the median of first egg dates from renesting attempts; average lay date was the median of first egg dates from all attempts – thus corresponding to the standard phenological index used in most studies.

#### Termination date index

The time when pairs cease to initiate renests following nesting failure provides an index of the end of the breeding season. In each year we modeled the probability of a failed pair renesting rather than terminating breeding activity, as a function of failure date, using a series of generalized linear models with logit link function and binomial error structure. Data were excluded from nests in which: (a) pair bonds were disrupted by divorce or mortality; (b) failure date was before the median lay date of first attempts; and (c) the failed attempt was located close to the field site boundary and pairs were suspected to be renesting outside the study area. All of these annual models of termination date had high explanatory power (mean McFadden's *r*^*2*^ = 59%) and were statistically significant (*P* < 0.05) in all but 1 year in which *r*^2^ = 85% (Table S1). Within each year, we used the predicted date at which 75% of breeding pairs did not renest as an index of annual termination date as this reflected the time when a large proportion of birds had stopped breeding and was robust to the inclusion of very late-terminating outliers, which only occur in some years. The 75% threshold termination date (henceforth ‘termination index’) was strongly correlated with the alternative termination thresholds of 50% (*rp*_13_ = 0.860, *P <* 0.001) and 90% (*rp*_13_ = 0.957, *P <* 0.001).

#### Breeding season length index

We calculated two indices of breeding season length. The first was the interval between the median initiation date and termination index for each year, which were not correlated with each other (*rp*_13_ = 0.16, *P >* 0.1); the second was the interval between the 10th and 90th percentile of all known first egg dates within each year. These two indices were strongly correlated with each other (*rp*_13_ = 0.83, *P <* 0.001) and we used the latter in subsequent analyses because it is more routinely used (e.g., Evans et al. 2005; Møller et al. 2010).

### Predation indices

The timing and intensity of nest predation may influence the timing of renesting and termination, and consequently breeding season length (Fig. 1D). We therefore calculated two measures of predation timing to test for trends in predation patterns: (i) the annual time of predation, as the median date of all nest predation events; (ii) the annual time of predation of first nesting attempts, as the median date of predation events of first nesting attempts; these two indices of predation timing were highly correlated (*rp*_14_ = 0.96, *P <* 0.0001) and we used the former index in subsequent analyses as it offered a more complete picture of predation timing. We also calculated two indices of predation intensity: (i) annual proportion of nests predated, which was the annual proportion of nests predated among nests known to have been predated or fledged; (ii) annual Mayfield predation estimate, which was the annual Mayfield estimate of predation risk throughout the nesting cycle; these two indices of predation intensity were highly correlated (*rp*_14_ = 0.98, *P* < 0.0001) and we used the latter in subsequent analyses because it offers a more comprehensive measure of predation rates (Mayfield 1975). Annual Mayfield estimates were calculated via a three-part process: (i) we calculated daily nest survival rates at the egg laying, incubation, and chick-rearing stages using the Mayfield method (Mayfield 1975); (ii) we used these daily survival rates to calculate the probability of a nest surviving the entire duration of each stage by raising the daily rates to the power of the stage duration in days, assuming stage durations of 9 days for egg laying, 15 days for incubation, and 16 days for chick rearing (the typical durations of these stages in the focal population; Hatchwell et al. 2004); (iii) we calculated annual nest predation risk as the product of the three annual stage-specific survival probabilities, subtracted from one.

### Assessing temporal trends

All statistical analyses were conducted in R, version 2.11.1 (R Development Core Team 2010). We first assessed trends in local mean monthly spring temperature and precipitation, using year as a predictor (both linear and squared terms); trends were assessed over two time periods: 1995–2011, that is, the duration of the focal study, and 1968–2010, that is, the period over which long-tailed tit phenology was analyzed at the national scale. We then investigated temporal trends in breeding events and predation, again using the linear and squared year terms as predictors and regressing them against each separate phenological index (initiation date, renesting date, average lay date, termination date index, and breeding season length index) and against each predation index (predation timing and predation intensity).

### Mechanisms of phenological change: climate, predation, food, adaptation

At the study site, long-tailed tit pairs typically start nest-building in February/March and egg laying in March/April; renesting attempts occur between March and May and pairs finish breeding by early June (MacColl and Hatchwell 2002). Therefore, to investigate the effects of climate on reproductive phenology we modeled: (a) initiation date in response to temperature and precipitation during February, March, and April; (b) renesting date in response to temperature and precipitation during March, April, and May, and the timing and intensity of predation; (c) termination index in response to temperature and precipitation during March, April, and May, and the timing and intensity of predation; and (d) breeding season length in response to temperature and precipitation during February, March, April, and May, and the timing and intensity of predation. We constructed multiple linear regression models with normal error structure (Shapiro–Wilk normality tests: *P* > 0.3 for all response variables). We used an information-theoretic approach to model selection in which all possible models were constructed given the set of predictors; model fit was assessed using Akaike's Information Criterion corrected for small sample size (AICc) and model averaging was conducted over the 95% confidence set of models (Burnham and Anderson 2002). Collinearity between climatic predictors was within the tolerance levels to which Information Theoretic methods are robust (Variance Inflation Factor <3.9 for all variables; Freckleton 2010; Table S2).

In order to test the hypothesis that the timing of breeding termination is influenced by the seasonal decline in caterpillar availability, we estimated the timing of peak caterpillar abundance in the Rivelin study site for the period 2009–2012, and determined the correlation between annual peak date and mean temperature during March and April. Full methods are described in Appendix S1.

Finally, we tested the hypothesis that local genetic adaptation may contribute to the observed changes in breeding phenology in the study population, because selection may act on the focal phenological traits (initiation and termination). Following the results of the climatic analysis, we modeled initiation date in response to March temperature and termination index in response to April temperature, comparing models in which year was included as an additive or interactive effect, in order to assess whether the observed temperature-phenology reaction norms have changed significantly over time. Such a temporal change in reaction norms would be compatible with the hypothesis that selection is driving evolutionary change in the form of phenological responses.

### Predicting phenology at larger spatial and temporal scales

Comparisons between local (i.e., Rivelin Valley) and national (i.e., U.K.) phenological responses could only be conducted using population average lay date calculated across all attempts, as other phenological indices are unavailable at the national scale. We first compared climatic models of long-tailed tit average lay date at the national and local scales, using data from 1995 to 2010, that is, the duration over which both local and national data were available. Second, we used the local climatic model of phenology to predict phenology at the national scale across a much larger time scale, that is, from 1968 to 2010, and regressed these predicted national annual lay dates against the national annual mean lay dates observed in the BTO dataset. If local climatic models of avian phenological responses can be scaled up to larger spatial and temporal scales we predict that the slope of this relationship will approximate unity. We assessed whether the performance of the model deteriorates further back in time, by calculating the square of the difference between predicted and observed values and regressing this against year (linear and quadratic terms; Piñeiro et al. 2008). We also assessed the performance of the locally derived model over years entirely outside the temporal span of the locally derived model (i.e., 1968–1994). In order to conduct a conservative test, March temperature was the only climatic variable used in these analyses as all other climatic variables had little influence on the average lay date of the Rivelin population (see Results).

## Results

### Extent of climatic change

Although spring mean monthly temperature and precipitation variables within the study region varied substantially between years, temporal trends within the study period (1995–2011, excluding 2001) were limited. The exception was April temperature which increased linearly (+0.12°C per year, *r*^2^_1,14_ = 0.22, *P* = 0.06; Table S3). From 1968 to 2010 all spring mean monthly temperatures increased linearly; the increase was most marked in April (+0.05°C per year, *r*^2^_1,40_ = 0.30, *P* < 0.001; Table S3). Temporal trends in spring mean monthly precipitation between 1968 and 2010 were negligible (Table S3).

### Temporal trends in phenological indices and predation

The range of annual median lay dates across all nesting attempts was from 2 to 21 April (mean 10 April ± 1.2 SE days), across first attempts it was from 29 March to 20 April (mean 8 April ± 1.4 days), and across renests it was from 17 April to 3 May (mean 25 April ± 1.4 days). The range of the annual termination index was from 22 April to 10 May (mean 30 April ± 1.5 days), and the breeding season length index, measured as the interval between the 10th and 90th percentile of all first egg lay dates, was from 13 to 33 days (mean 24 ± 1.3 days).

**Figure 3:**
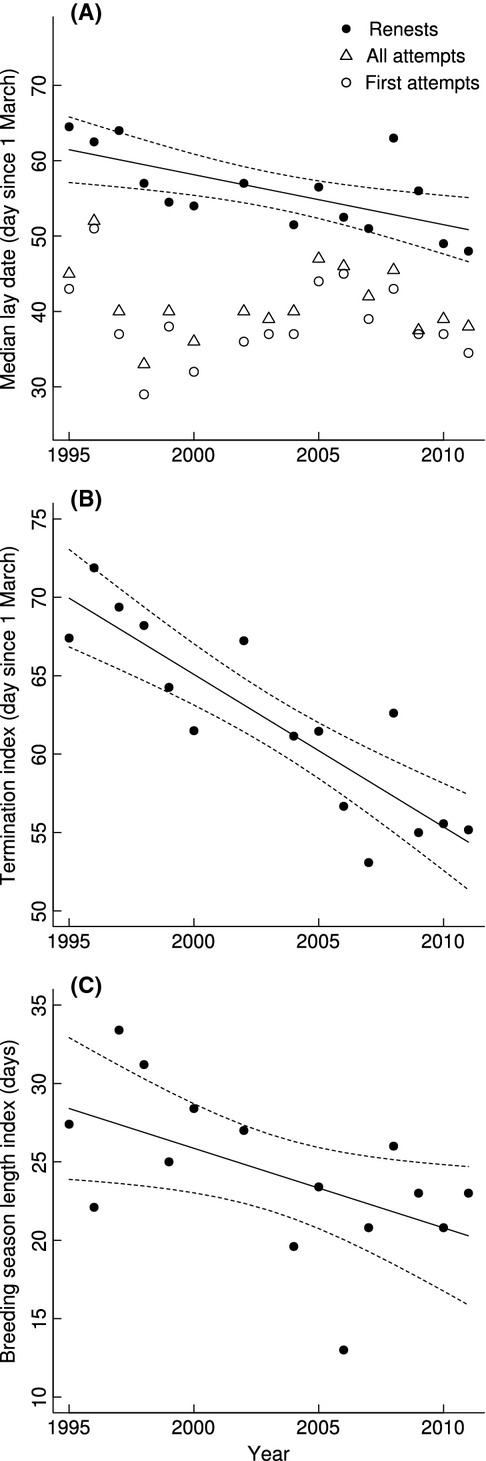
Temporal trends in long-tailed tit phenology in the Rivelin Valley, Sheffield (1995–2011), showing linear regressions (solid lines) ± 95% CI (dashed lines). (A) annual median lay dates of renests have advanced (slope = −0.66 ± 0.21, *r*^2^ = 0.44), while annual median lay dates of all nesting attempts and first attempts show no significant change; (B) timing of termination has advanced (slope = −0.97 ± 0.15, *r*^2^ = 0.77); and (C) breeding season length has decreased (slope = −0.50 ± 0.22, *r*^2^ = 0.30). Day 1 represents 1 March.

There was a linear trend toward advancing lay date over the period 1995–2011 for all three nesting categories (i.e., all attempts, first attempts, and renests), but this was significant only for renests, with the fitted model predicting an advance of 0.66 days per annum (*r*^2^_1,13_ = 0.44, *P* < 0.01; Fig. 3A, Table 1). Termination date showed a significant linear advance from 1995 to 2011 of 0.97 days per annum (*r*^2^_1,13_ = 0.77, *P* < 0.0001; Fig. 3B, Table 1). The breeding season length index exhibited a linear reduction of 0.51 days per annum (*r*^2^_1,13_ = 0.30, *P* < 0.05; Fig. 3C, Table 1), equating to a 33% reduction in the average length of the reproductive window.

**Table 1:** Temporal trends in phenological indices (initiation date i.e., average lay date of first attempts, renesting date, average lay date, termination index, and breeding season length index), indices of predation timing (median predation date, median predation date of first attempts) and indices of predation intensity (proportion of nests predated, Mayfield estimate of predation risk), in the Rivelin Valley, Sheffield (1995–2011)

Index	Linear trend (days ± 1SE)	*r*^2^	*F*_df_	*P-*value	Linear model ΔAICc_null_
Initiation date	−0.10 ± 0.28	0.01	0.14_1,14_	0.72	+3.02
Renesting date*	−0.66 ± 0.21	0.44	10.07_1,13_	0.007	−5.42
Average lay date	−0.12 ± 0.24	0.02	0.26_1,14_	0.62	+2.89
Termination index*	−0.97 ± 0.15	0.77	42.50_1,13_	<0.0001	−18.59
Breeding season length index*	−0.51 ± 0.22	0.30	5.47_1,13_	0.04	−2.09
Predation date	0.03 ± 0.37	<0.01	0.01_1,14_	0.93	+3.17
Predation date of 1st attempts	0.07 ± 0.46	<0.01	0.02_1,14_	0.88	+3.16
Proportion of nests predated	−0.007 ± 0.005	0.14	2.28_1,14_	0.15	+0.77
Mayfield estimate of predation	0.008 ± 0.005	0.15	2.48_1,14_	0.14	+0.58

Linear and quadratic models were compared for each response variable by assessing the change in Akaike's Information Criterion corrected for small sample size (AICc) with respect to the null model, where a negative ΔAICc_null_ indicates evidence of a temporal trend; linear models were always more parsimonious than quadratic ones, and linear trends are therefore displayed with associated statistics.

* denotes variables showing evidence of a temporal trend (*P* < 0.05 and negative ΔAICc_null_).

Predation showed no temporal trend, whether calculated over all predation events or just predation of first nesting attempts (Table 1). Similarly, neither index of predation intensity showed a temporal trend (Table 1), despite substantial variation between years (annual proportion of predations ranged from 0.52 to 0.85, mean ± 1SE = 0.72 ± 0.10; annual Mayfield estimates ranged from 0.47 to 0.80, mean ± 1SE = 0.67 ± 0.10; Table S4).

### Effects of climate, predation, and caterpillar phenology on avian phenology

**Figure 4:**
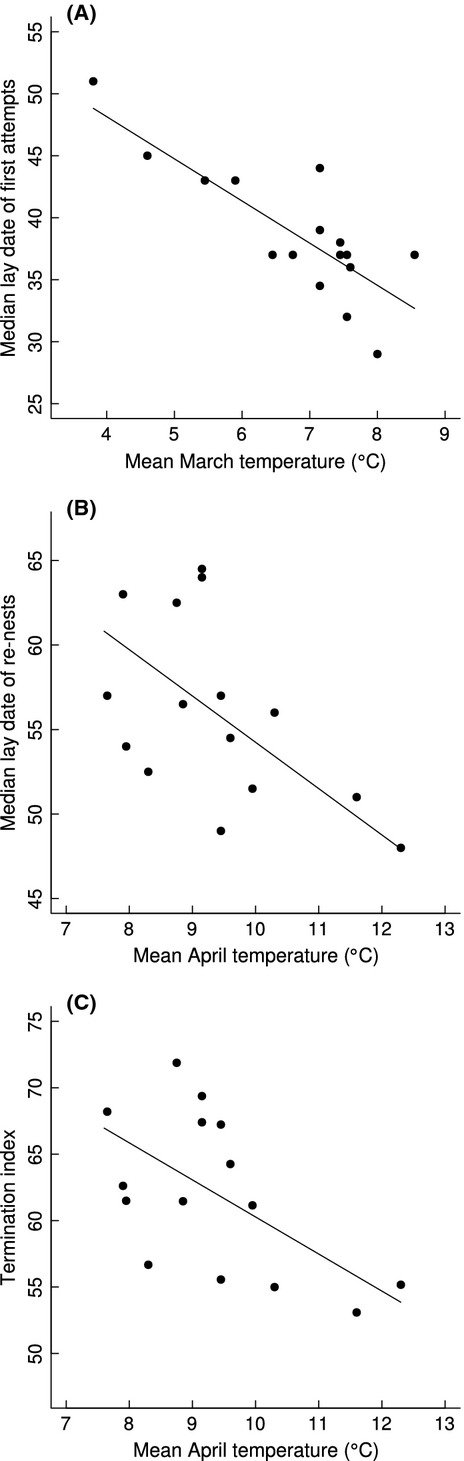
Associations between climate and long-tailed tit phenology in the Rivelin Valley, Sheffield (1995–2011), showing relationships from model averaged estimates holding other variables at mean values. (A) median lay date of first attempts advances with warmer March temperature (slope = −3.40 ± 0.74, partial *r*^2^ = 0.58); (B) median lay date of renest attempts advances with warmer April temperature (slope = −2.74 ± 1.18, partial *r*^2^ = 0.35); (C) termination date advances with warmer April temperature (slope = −2.79 ± 1.21, partial *r*^2^ = 0.32). Day 1 represents 1 March.

Climate explained a consistently large proportion, between one-third and two-thirds, of the annual variation in long-tailed tit phenological indices. Warm March temperatures advanced the median lay date of all attempts (model averaged partial *r*^2^ = 0.54, β = −2.93, *n* = 15) and the median lay date of first attempts (model averaged partial *r*^2^ = 0.58, β = −3.44, *n* = 15; Fig. 4A). All other climatic variables had little influence on these phenological indices (Table 2). Median lay dates of renests advanced in years with warm Aprils (model averaged partial *r*^2^ = 0.35, β = −2.74, *n* = 14; Fig. 4B); there was also a marginal tendency for wet Aprils to advance the timing of renests (model averaged partial *r*^2^ = 0.06, β = −0.03, *n* = 14); all other climate variables, including March temperature, and the timing and intensity of predation had little influence on renesting dates (Table 2).

**Table 2:** Model averaging results from multiple regressions of breeding phenology (median lay date of all attempts/first attempts/renest attempts, termination index, and breeding season length index) in response to monthly spring temperature (temp) and precipitation (prec) during 1995–2011. Predation (pred) intensity and timing were also included as predictors in the latter three models

Model	February temp	March temp	April temp	May temp	February prec	March prec	April prec	May prec	Pred intensity	Pred timing	Model average *r*^2^
All attempts											
Estimate	−0.03	−2.93	−0.01	−0.16	<0.01	−0.01	<0.01	<0.01	n/a	n/a	
±1SE	0.22	0.72	0.25	0.54	0.01	0.02	0.01	0.01	n/a	n/a	
Partial *r*^2^	<0.01	0.54	<0.01	<0.01	0.01	0.01	<0.01	<0.01	n/a	n/a	**0.65**
1st attempts											
Estimate	−0.14	−3.4	−0.01	n/a	<0.01	−0.01	−0.01	n/a	n/a	n/a	
±1SE	0.39	0.74	0.26	n/a	0.01	0.02	0.01	n/a	n/a	n/a	
Partial *r*^2^	0.01	0.58	<0.01	n/a	<0.01	<0.01	0.01	n/a	n/a	n/a	**0.69**
Renest attempts											
Estimate	n/a	0	−2.74	0	n/a	0	−0.03	0	+3.34	0	
±1SE	n/a	0	1.18	0	n/a	0	0.04	0	8.79	0	
Partial *r*^2^	n/a	0	0.32	0	n/a	0	0.06	0	0.02	0	**0.35**
Termination											
Estimate	n/a	0	−2.79	0	n/a	−0.02	0	0	0	0	
±1SE	n/a	0	1.21	0	n/a	0.05	0	0	0	0	
Partial *r*^2^	n/a	0	0.32	0	n/a	0.02	0	0	0	0	**0.33**
Breeding season length											
Estimate	+1.32	+0.76	−0.60	−0.13	<0.01	−0.01	<0.01	−0.02	+0.02	−0.04	
±1SE	1.1	1.21	0.95	0.59	0.01	0.02	0.01	0.04	1.63	0.12	
Partial *r*^2^	0.24	0.10	0.05	0.01	<0.01	0.01	<0.01	0.04	<0.01	0.02	**0.5**

Variables not included are indicated by n/a. Variables not retained in the model average are indicated by 0.

The index of termination date advanced in years with warm Aprils (model averaged partial *r*^2^ = 0.32, β = −2.79, *n* = 14; Fig. 4C); other climate variables, including March temperature, and the timing and intensity of predation had little influence (Table 2). Breeding seasons were longer in years with warm February (model averaged partial *r*^2^ = 0.24, β = +1.32, *n* = 14) and March temperatures (model averaged partial *r*^2^ = 0.10, β = +0.76, *n* = 14), and there was evidence that they were reduced in years with warm Aprils (model averaged partial *r*^2^ = 0.05, β = −0.6, *n* = 14; Table 2). All other climatic variables and the timing and intensity of predation had little influence on breeding season length (Table 2).

Caterpillar abundance peaked earlier in years with warmer April temperatures over the period 2009–2012 (*rp* = −0.72); data were insufficient to estimate reliably the slope and statistical significance of this relationship (*n* = 4), but the observed relationship equates to a strong effect size (Cohen 1988). Peak caterpillar date showed no relationship with March temperature (*rp* = 0.05, *n* = 4).

### Microevolution versus phenotypic plasticity

There was a slight tendency for the relationship between March temperature and timing of initiation to become weaker over time, and for the relationship between April temperature and timing of termination to become stronger over time, but neither of these trends were significant (2-way ANOVA comparing additive ‘*temperature + year*’ with interactive ‘*temperature* × *year*’ models: Initiation: *F*_1_ = 0.172, *SS* = 2.00, *P* = 0.69; Termination: *F*_1_ = 0.002, *SS* = 0.017, *P* = 0.97).

### Predicting phenology at larger spatial and temporal scales

**Figure 5:**
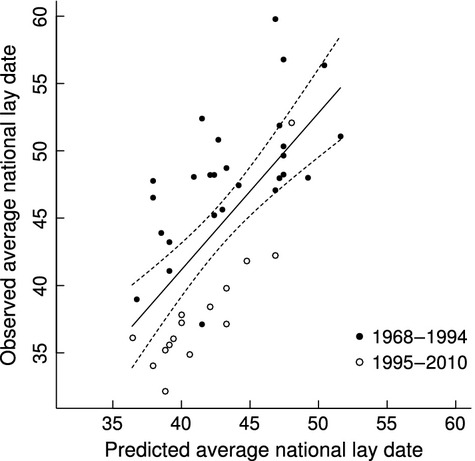
Predictions of national average lay date derived from the local climatic model (average lay date in response to March temperature), compared with observed national average lay dates, 1968–2010 (filled circles: 1968–1994; open circles: 1995–2010). Observed and predicted dates are strongly correlated (solid line: *r*_40_ = 0.68; *P* < 0.001), and the slope of this relationship is close to unity (1.17 ± 0.20); dashed lines represent 95% confidence intervals. Predictive capacity is similar when restricting prediction to those years that were not represented in the local model (i.e., 1968–1994; filled circles; *r*_25_ = 0.63; *P* < 0.001). Day one represents 1 March.

The form of bivariate relationships between long-tailed tit phenology and March temperature during 1995–2010 at the local scale (β = −2.94; 95% confidence intervals −4.29 to −1.58) was similar to that at the national scale (β = −3.62; 95% CIs −4.94 to −2.31). Moreover, predictions of national mean lay date from 1968 to 2010, derived from the climatic (March temperature) model of the local Rivelin population's phenological response during 1995–2011, were strongly correlated with the observed values of the national population's mean lay date (*P* < 0.001; *r*_40_ = 0.678), and the slope of the relationship between predicted and observed values was very close to unity (β = 1.17; 95% CIs 0.76–1.57; Fig. 5). Predictive capacity was still high when using the local phenology model to predict national phenology in years entirely outside the range of years during which local data were collected, that is, from 1968 to 1994 (*P* < 0.001; *r*_25_ = 0.629), and the slope between predicted and observed values in this period was lower, but again not significantly different from unity (β = 0.76; 95% CIs 0.37–1.14; Fig. 5). Predictive capacity does not deteriorate over time (linear model of the squared difference between predicted and observed values, regressed against year: *F*_1,40_ = 1.48, *r*^2^ = 0.04, *P* = 0.23; quadratic model: *F*_2,39_ = 0.88, *r*^2^ = 0.04, *P* = 0.42), although, the extent of advance in mean lay dates tends to be under predicted from the March temperature model in recent years (1995–2011; Fig. 5). The range of March temperatures used when constructing the local model (1995–2011: 3.80–8.6°C) was similar to the range of March temperatures experienced by the national population over both time periods (1968–2010: 3.3–8.4 °C; 1968–1994: 3.3–8.3°C). The residuals of the relationship between observed and predicted national annual mean lay dates were not associated with temperature (1968–2010: *P* > 0.05, *r*_40_ < 0.0001; 1968–1994: *P* > 0.05, *r*_25_ < 0.0001).

## Discussion

This study shows that the standard phenological index of mean population lay date does not pick up key phenological responses that could have important demographic impacts, such as the 33% reduction in breeding season length observed here. More positively, we show that local phenological models can be successfully applied at larger spatial and temporal scales. This ability to scale up from local phenological models has rarely been demonstrated previously (but see Hodgson et al. 2011), and we are not aware of any such avian studies. We thus offer novel insight into an approach through which detailed information from intensive local studies can be used to develop broader scale predictive models of climate change impacts.

### Scaling up from local to national, short-term to long-term

The local and national models of lay date as a function of March temperature are statistically indistinguishable, and the locally derived phenology model performs reasonably well in predicting previous mean lay dates at the national scale. We thereby show that intensive local studies that capture a broad range of phenological responses can provide useful inference at much larger spatial scales. Such spatial extrapolation is likely to be crucial for developing broad-scale predictive capacity in the face of climate change, because the inherent patchiness of phenological data at national and regional scales mean that such broad-scale data offer limited ability to detect patterns such as the divergent trends in breeding initiation and termination. It is important to note that there are inevitably limits to such spatial extrapolations, particularly in species with broad geographic ranges, such as the long-tailed tit. Applying local models to areas experiencing markedly different annual weather regimes would clearly be inappropriate, and multiple local studies in areas of contrasting climates are thus required in order to develop predictions across the species' large geographic range. However, this study shows that local studies can be used to develop accurate phenological predictions at much broader spatial scales within regions experiencing similar climatic regimes. Given the key role of phenology in determining species' responses to climate change (Miller-Rushing et al. 2010), this is an essential first step towards the goal of developing mechanistic models of species' responses to future climate change (Diez et al. 2012).

Furthermore, the local model's predictive capacity was upheld when applied to a much longer and non-overlapping time period than that used to construct the model, and predictive capacity did not decrease further back in time or at higher temperatures. This indicates that temporal extrapolation from relatively short-term studies (17 years in this case) is possible. Much caution is needed when make predictions outside the range of climatic conditions experienced during the reference study. This is not a major issue in our study, however, as the range of climatic conditions used in the local model encompasses most of the predicted range of future climatic conditions across the U.K. over the next 60 years under a high carbon emissions scenario (March: used in model 3.8–8.6°C, predicted 6–9°C; April: used in model 7.7–12.3°C, predicted 7–12°C; Jenkins et al. 2008). Although the increasing discrepancy between historic and future climatic variables further into the future will make longer-term predictions tricky, our analysis strongly suggests that data from intensive local studies can be used to predict future phenological shifts over time scales of several decades.

### Phenological indices and potential demographic impacts

The standard phenological index of mean population lay date did not detect key phenological responses that could have important demographic impacts. The local population's mean lay date and initiation date showed no temporal trend, but timing of renesting and breeding termination have advanced significantly. This has led to an 8-day contraction of the breeding season length index, which equates to a 33% loss of the average reproductive window. This change may have little effect if late broods are unproductive, but in long-tailed tits, fledglings from late broods are as likely to recruit into the breeding population as those from broods raised earlier in the season (Sharp et al. 2008), perhaps partly because later broods are more likely to gain benefits from helpers in this cooperatively breeding species (MacColl and Hatchwell 2004). In many non-cooperatively breeding species, late broods have greater fitness in some years (e.g., guillemots *Uria aalge*: Harris et al. 1992; great tits *Parus major*: Monrós et al. 2002), or are typically essential for maintaining positive population growth rates (Green 1988; Farnsworth and Simons 2001; Grzybowski and Pease 2005; Podolsky et al. 2007; Wright et al. 2009). A decline in breeding season length over recent years has been observed in several single-brooded species across Denmark, despite increases in the duration of breeding seasons in several multi-brooded species (Møller et al. 2010). This suggests that single-brooded species may experience stronger environmental constraints on breeding season length than multi-brooded species, at least in populations at the lower end of their thermal optimum. Given the recent observed decline in the incidence of double brooding in Dutch great tits (Husby et al. 2009), and the results of this study, it seems likely though that breeding season durations in both single and multi-brooded species may become climatically constrained in the future.

The observed discrepancy between rates of advance in initiation and termination in the local study probably also explains the observation that in recent years (1995–2011) national mean lay dates have advanced more than predicted from March temperature alone (Fig. 5). The observed national advance is likely to be due partly to an advance in termination date due to warmer Aprils, rather than solely to advancing initiation due to warmer March temperatures; indeed, between 1995 and 2010 April temperatures increased nationally, whereas March temperatures showed no significant change (HadCET, Parker et al. 1992). Given that such an advance in timing of termination at the national scale could have drastic demographic consequences, we show that assessments of species' sensitivity to climate change based on average population lay date are inadequate. In addition to the scaling-up approach described above, we suggest that assessments based on broad-scale phenological data should incorporate information on the variance in timing of breeding, as well as simply the average timing. Such practice is currently rare, but would be straightforward even with existing data from national nest monitoring schemes (as demonstrated in North American tree swallows *Tachycineta bicolor*, Winkler et al. 2002). This study showed the 10th–90th percentile of first egg dates to be a good indicator of breeding season length; although national monitoring data is inevitably less comprehensive than the data presented here, we suggest that the use of a similar metric at the national scale would add an important dimension to phenological monitoring that could enable earlier detection of future ecological problems arising from climate change.

### Microevolution versus phenotypic plasticity

There was no change in the form of the reaction norms between temperature and phenology (initiation and termination) in the Rivelin population over the 17 years of our study. This suggests that the majority of observed phenological change was due to phenotypic plasticity rather than selection pressure resulting in genetic adaptation. Lay date is a heritable trait, with passerine heritability estimates typically in the range of 0.16–0.45 (Sheldon et al. 2003), and selection pressures can generate divergence in breeding time reaction norms across conspecific populations (Caro et al. 2009; Gienapp et al. 2010). Evidence for microevolutionary change in breeding data is, however, very rare (Gienapp et al. 2008); indeed, it is often lacking even in studies that have clearly demonstrated heritability of lay dates (Sheldon et al. 2003; Gienapp et al. 2006). Local adaptation of breeding time in response to climate may, however, be more prevalent than currently believed (Gienapp et al. 2008). This could limit the predictive capacity of local phenological models at broader spatial scales, given that populations can experience different selection pressures (Visser et al. 2003; Caro et al. 2009; Gienapp et al. 2010). Further work is required to quantify the extent to which microevolutionary change contributes to phenological shifts, but we provide initial evidence that phenotypic plasticity is more important than genetic change in our focal long-tailed tit population.

### Mechanisms of phenological change

The climatic model of local long-tailed tit phenology explains the majority (65%) of the temporal variation in the population's mean lay date, with March temperature being the most important driver. Earlier breeding in years with warm March temperatures is likely to be driven largely by the alleviation of energetic and resource constraints; this topic has received copious attention in the literature and we therefore do not discuss it further here (e.g., Crick and Sparks 1999; Visser et al. 2002; Schaper et al. 2011; Vatka et al. 2011). We have also presented rare evidence that the termination of breeding is highly sensitive to temperature, with breeding ending earlier in years with warm April temperatures (Fig. 3C). One plausible mechanism for this is an influence of April temperature on food availability. Caterpillars are the dominant component of long-tailed tit chick diets (Cramp and Perrins 1993; P. Gullett, pers. obs.) and the optimum nestling food source in terms of nutrition and energetic value (Visser et al. 2006; García-Navas and Sanz 2011). Given that temporally matching breeding with the peak in caterpillar abundance benefits productivity and survival in ecologically similar species (van Noordwijk et al. 1995; Thomas et al. 2001), we hypothesized that the timing of the seasonal decline in caterpillar availability may be an important determinant of the timing of breeding termination. Our limited data suggest that caterpillar abundance at the study site peaked earlier in years with warmer April temperature, but showed no relationship with March temperature. April temperature thus seems to have a similar influence on the timing of peak caterpillar abundance at our study site to that reported in other UK woodlands, in which peak frass biomass advances by 8.5 days per 1°C increase in spring temperature (Smith et al. 2011). Applying this relationship with our study site, in which April temperatures have increased by 1.9°C over the course of this study, indicates that if long-tailed tits track caterpillar phenology there should be a 17-day advance in breeding termination. This predicted advance in termination date is remarkably close to the observed advance of 16 days. It thus seems likely that earlier peaks in caterpillar abundance in years with warm Aprils contribute to the earlier termination of long-tailed tit breeding in these years. Earlier seasonal decline in caterpillar availability in warm years is thought to contribute to a decline in double brooding of great tits (Husby et al. 2009), and earlier gonadal regression in warmer years caused earlier cessation of breeding in an aviary study of starlings *Sturnus vulgaris* (Dawson 2005). There is thus mounting evidence that climate change is driving an advance in the timing of breeding termination in numerous species, and further study into the mechanisms behind this should be a priority for research.

Climate explained half the variation in breeding season length in our study population, primarily due to increased duration in years with warm February and March temperatures and shortening in years with warm Aprils. The effect of February temperature was unexpected given that February temperature was not closely associated with the timing of first breeding attempts. This pattern could partly arise because February and March temperatures are positively correlated, but the tolerance levels were sufficiently low to justify including both variables in the analysis. We consider it likely that warmer conditions in the prebreeding period may enhance parental body condition through reducing energetic expenditure for thermoregulation and food acquisition (e.g., Crick and Sparks 1999; Visser et al. 2002; Schaper et al. 2011; Vatka et al. 2011), enabling prolonged investment in energetically demanding reproductive behavior.

Finally, we found no evidence for a trend in the timing or intensity of nest predation in this species, and the breeding phenology was not associated with either predation parameter. In this study predation patterns are not a primary driver of breeding phenology, but species in different ecosystems or locations could be more susceptible to changing predation patterns (Adamik and Král 2008) and predation effects should therefore be taken into account in phenological monitoring schemes.

## Conclusion

In conclusion, we have shown that climatic models of phenological responses derived from intensive local studies can scale-up to predict responses at much larger spatial and temporal scales. We show that current patterns of climate change promote earlier termination of breeding (which is associated with earlier declines in food availability), despite little change in the timing of breeding initiation. This results in potential reproductive capacity being significantly reduced. These trends are not detected by the routinely used phenological indicator of population mean lay date, demonstrating that the choice of phenological metric can bias estimates of species' sensitivity to climate change. The observed phenological shifts appear to have arisen primarily from phenotypic plasticity rather than microevolutionary change. Developing predictive phenological models using indices that capture a more complete spectrum of phenological shifts is of fundamental importance to develop mechanistic models of species' vulnerability to future climatic change.

## Supporting Information

**Table S1.** Annual models of renesting probability (1995–2011).

**Table S2.** Correlation coefficients between weather variables (1995–2011).

**Table S3.** Range and temporal trends in weather variables (1968–2011).

**Table S4.** Estimates of annual and stage-specific predation intensity (1995–2011).

**Appendix S1.** Methods used to assess the seasonal pattern of caterpillar abundance (2009–2012).
