# Supplementary material for: Phenological indices of avian reproduction: cryptic shifts and prediction across large spatial and temporal scales
Source: Ecol Evol. 2013 May 21;3(7):1864–77. doi: 10.1002/ece3.558 (PMC3728930; doi:10.1002/ece3.558)
Supplement: Supplementary file 1 [file ece30003-1864-SD1.doc]

SUPPORTING INFORMATION

**Table S1.** Logistic regressions of re-nesting occurrence (re-nest vs termination) following nest failure, in response to failure date, for each year of the study used in analysis of termination date. McFadden’s *r*^2^ was calculated as the difference in deviance between the null and explanatory model, divided by the deviance of the null.

| Year | n | Parameter estimate ±1SE | Wald statistic | *P* value | McFadden's *r*^2^ | Dispersion parameter |
| --- | --- | --- | --- | --- | --- | --- |
| 1995 | 19 | -0.19±0.09 | 2.01 | 0.045 | 0.39 | 0.95 |
| 1996 | 40 | -0.26±0.09 | 2.87 | 0.004 | 0.62 | 0.56 |
| 1997 | 51 | -0.13±0.04 | 3.76 | <0.001 | 0.38 | 0.89 |
| 1998 | 36 | -0.01±0.03 | 2.89 | 0.004 | 0.35 | 0.95 |
| 1999 | 38 | -0.02±0.05 | 3.24 | 0.001 | 0.47 | 0.77 |
| 2000 | 33 | -0.13±0.04 | 2.96 | 0.001 | 0.40 | 0.88 |
| 2002 | 61 | -0.12±0.03 | 3.82 | <0.001 | 0.33 | 0.95 |
| 2004 | 57 | -0.33±0.11 | 2.97 | 0.003 | 0.70 | 0.43 |
| 2005 | 56 | -0.17±0.04 | 3.91 | <0.001 | 0.58 | 0.58 |
| 2006 | 44 | -0.42±0.21 | 2.07 | 0.038 | 0.90 | 0.14 |
| 2007 | 53 | -0.24±0.07 | 3.26 | 0.001 | 0.74 | 0.35 |
| 2008 | 75 | -0.37±0.12 | 3.14 | 0.002 | 0.88 | 0.37 |
| 2009 | 33 | -1.19±0.91 | 1.30 | 0.190 | 0.85 | 0.17 |
| 2010 | 52 | -0.69±0.36 | 1.93 | 0.050 | 0.66 | 0.20 |
| 2011 | 57 | - 0.29±0.09 | 3.39 | <0.001 | 0.66 | 0.48 |

**Table S2.** Pearson correlation coefficients between spring temperature (temp) and precipitation (prec) between 1995 and 2011 in Sheffield, UK that are used in regressions of long-tailed tit phenology. Data from 2001 are excluded as avian data are excluded from this year. * denotes correlations that are significant at *P* < 0.05 (*n* = 16). Note that all collinearity between climatic predictors was within the limits to which the information theoretic approach is robust (Freckleton 2010), with Variance Inflation Factor < 3.9 for all variables.

|  | March temp | April temp | May temp | Feb prec | March prec | April prec | May prec |
| --- | --- | --- | --- | --- | --- | --- | --- |
| Feb temp | 0.559* | 0.026 | 0.534* | 0.275 | -0.590 | 0.234 | 0.057 |
| March temp |  | 0.212 | 0.569* | 0.046 | -0.408 | 0.219 | -0.133 |
| April temp |  |  | 0.120 | 0.210 | -0.609* | -0.606* | 0.059 |
| May temp |  |  |  | -0.163 | 0.188 | 0.319 | -0.010 |
| Feb prec |  |  |  |  | -0.512* | -0.378 | -0.010 |
| March prec |  |  |  |  |  | 0.313 | 0.160 |
| April prec |  |  |  |  |  |  | -0.170 |

**Table S3.** Range and temporal trends in monthly mean temperature (temp) and total precipitation (prec) at Weston Park Weather Station (5km from the Rivelin Valley), from 1995-2011 (the period of this study) and 1968-2010 (the period over which national trends in long-tailed tit phenology are recorded). Linear and quadratic models were compared for each weather variable by assessing the change in AICc with respect to the null model, where a negative ΔAICc_null_ indicates evidence of a temporal trend; quadratic models were never more parsimonious than the null, and linear trends are therefore displayed below with associated statistics. * denotes variables showing evidence of a temporal trend (p<0.1 and negative ΔAICc_null_). All data from 2001 are excluded to maintain consistency with long-tailed tit analyses.

| Weather variable | Time period | Month | Range | Linear trend  ±1SE | *r*^2^ | *F*_df_ | *P* | Linear model ΔAICc_null_ |
| --- | --- | --- | --- | --- | --- | --- | --- | --- |
| Temp (ºC) | 1995-2011 | February | 2.30 – 8.15 | -0.08±0.08 | 0.07 | 0.99_1,14_ | 0.34 | +1.99 |
|  |  | March | 3.80 – 8.55 | +0.01±0.07 | <0.01 | 0.03_1,14_ | 0.86 | +3.04 |
|  |  | April* | 7.65 – 12.30 | +0.12±0.06 | 0.22 | 4.04_1,14_ | 0.06 | -0.98 |
|  |  | May | 9.15 – 13.25 | +0.04±0.05 | 0.05 | 0.70_1,14_ | 0.42 | +2.29 |
|  | 1968-2010 | February* | -1.00 – 8.15 | +0.05±0.02 | 0.12 | 5.59_1,40_ | 0.02 | -3.16 |
|  |  | March* | 2.35 – 8.85 | +0.05±0.02 | 0.20 | 10.08_1,40_ | 0.003 | -7.11 |
|  |  | April* | 5.65 – 11.60 | +0.05±0.01 | 0.30 | 17.03_1,40_ | <0.001 | -12.57 |
|  |  | May* | 9.15 – 13.65 | +0.03±0.01 | 0.14 | 6.74_1,40_ | 0.01 | -4.21 |
| Prec (mm) | 1995-2011 | February | 9.3 – 173.9 | -1.15±1.98 | 0.02 | 0.34_1,14_ | 0.57 | +2.70 |
|  |  | March | 12.4 – 100.2 | -0.61±1.38 | 0.01 | 0.20_1,14_ | 0.66 | +2.85 |
|  |  | April | 5.8 – 153.3 | -1.97±2.09 | 0.06 | 0.89_1,14_ | 0.36 | +2.09 |
|  |  | May | 17.5 – 129.8 | +0.41±1.45 | 0.01 | 0.08_1,14_ | 0.78 | +2.99 |
|  | 1968-2010 | February | 4.6 – 201.4 | -0.28±0.51 | 0.01 | 0.31_1,40_ | 0.58 | +2.01 |
|  |  | March | 15.1 – 149.9 | -0.51±0.40 | 0.04 | 1.65_1,40_ | 0.21 | +0.64 |
|  |  | April | 5.8 – 153.3 | +0.005±0.45 | <0.01 | <0.001_1,40_ | 0.99 | +2.33 |
|  |  | May | 14.4 – 129.8 | -0.21±0.37 | 0.01 | 0.34_1,40_ | 0.57 | +1.98 |

**Table S4.** Two indices of predation intensity, and annual stage-specific predation risks calculated using the Mayfield method. 'Proportion of nests predated' is the total proportion of nests that were predated out of all nests known to have been predated or fledged. 'Mayfield predation estimate' is the total probability that a nest was predated at some point during the nesting period, calculated as the product of the three stage-specific risk estimates from the Mayfield method, which are displayed as 'Pred (egg/inc/chick)'; this overall 'Mayfield predation estimate' was used as the index of predation intensity in subsequent analyses. 'No. nest days' is the total number of days during which a nest was active, summed for all nests over the entire breeding season within each year, as used in calculations of Mayfield estimates.

| Year | Proportion of nests predated | Mayfield predation estimate | Pred (egg) probability | Pred (inc) probability | Pred (chick) probability | No. nest days |
| --- | --- | --- | --- | --- | --- | --- |
| 1995 | 0.65 | 0.59 | 0.04 | 0.43 | 0.26 | 764 |
| 1996 | 0.72 | 0.68 | 0.17 | 0.32 | 0.44 | 939 |
| 1997 | 0.85 | 0.79 | 0.29 | 0.55 | 0.32 | 919 |
| 1998 | 0.64 | 0.55 | 0.09 | 0.20 | 0.38 | 1005 |
| 1999 | 0.63 | 0.57 | 0.14 | 0.27 | 0.33 | 1279 |
| 2000 | 0.52 | 0.47 | 0.08 | 0.34 | 0.13 | 1495 |
| 2001 | -- | -- | -- | -- | -- | -- |
| 2002 | 0.79 | 0.75 | 0.14 | 0.50 | 0.42 | 1140 |
| 2003 | 0.68 | 0.61 | 0.10 | 0.22 | 0.45 | 1254 |
| 2004 | 0.59 | 0.54 | 0.06 | 0.25 | 0.34 | 2003 |
| 2005 | 0.83 | 0.80 | 0.10 | 0.26 | 0.71 | 1093 |
| 2006 | 0.73 | 0.66 | 0.04 | 0.31 | 0.49 | 1341 |
| 2007 | 0.84 | 0.78 | 0.10 | 0.25 | 0.67 | 1078 |
| 2008 | 0.81 | 0.76 | 0.16 | 0.45 | 0.49 | 1628 |
| 2009 | 0.71 | 0.66 | 0.18 | 0.28 | 0.43 | 1044 |
| 2010 | 0.77 | 0.68 | 0.04 | 0.27 | 0.54 | 1650 |
| 2011 | 0.77 | 0.76 | 0.26 | 0.46 | 0.40 | 1471 |
| mean | 0.72 | 0.67 | 0.12 | 0.33 | 0.43 | 1256.4 |
| sd | 0.10 | 0.10 | 0.08 | 0.11 | 0.14 | 326.4 |

**Appendix S1. Assessing caterpillar abundance**

The seasonal pattern of caterpillar abundance was assessed using the frass-fall method of Tinbergen (1960). During the long-tailed tit breeding seasons of 2009-2012, caterpillar abundance was estimated for 16 trees within the Rivelin study site, with two oak *Quercus sp.* and two birch *Betula sp.*) trees monitored at each of four sites located throughout the long-tailed tit study area. These are the two commonest tree species within the study site, and are frequently used by foraging long-tailed tits during the chick-rearing period. At each tree, we collected caterpillar frass falling from the canopy by placing a wooden frame (50 cm x 50 cm) covered in nylon mesh beneath the canopy centre. Sampling was only conducted during dry periods, as rain can wash frass out of the mesh, and when wind-speed was 10mph or less, to reduce the risk that some of the collected frass originated from nearby trees rather than the focal one. Due to these constraints sampling duration and frequency varied between sampling events, but on average sampling was conducted for a duration of two days at four-day intervals. Sampling commenced within a few days of bud burst and continued until the amount of frass collected was one quarter or less of the maximum amount collected; sampling was conducted most frequently when frass was abundant, to increase the accuracy of temporally pin-pointing the peak abundance. There were 11 sampling events in 2009, 2010 and 2012, and 12 in 2011. To separate frass from non-frass material, samples were passed through a series of soil sieves and sorted by hand. Frass was then dried for 24 hours at 80ºC, and weighed to an accuracy of 0.002g using a Tanita digital scale (model 1230, Tanita, Japan). Mean frass mass (hr^-1^) was converted to caterpillar biomass using the equation derived by Tinbergen & Diaz (1994):

Caterpillar biomass = (24.38 x F) – (0.767 x F x T)

where F = frass dry mass (mg) and T = ambient temperature (ºC).

Data on temperature during each sampling period was obtained from the Weston Park weather station, which is located 5km from the centre of the study site. The date of peak caterpillar abundance was estimated as the Julian date (1 March = day 1) of observed maximum caterpillar biomass.

**Supporting references**

Freckleton, R.P. (2010) Dealing with collinearity in behavioural and ecological data: model averaging and the problems of measurement error. *Behavioral Ecology and Sociobiology*, 65, 91–101.

Tinbergen, J.M. & Dietz, M.W. (1994) Parental energy expenditure during brood rearing in the great tit (Parus major) in relation to body mass, temperature, food availability and clutch size. *Functional Ecology*, 8, 563-572.

Tinbergen, L. (1960) The natural control of insects in pinewoods. I, Factors influencing the intensity of predation by songbirds. *Archives Neerlandaises de Zoologie*, 13, 265-343.
